# Extracellular vesicles isolated by size-exclusion chromatography present suitability for RNomics analysis in plasma

**DOI:** 10.1186/s12967-021-02775-9

**Published:** 2021-03-12

**Authors:** Yang Yang, Yaojie Wang, Sisi Wei, Chaoxi Zhou, Jiarui Yu, Guiying Wang, Wenxi Wang, Lianmei Zhao

**Affiliations:** 1 Research Center, The Fourth Hospital of Hebei Medical University, Shijiazhuang, 050011 Hebei China; 2 Department of General Surgery, The Fourth Hospital of Hebei Medical University, Shijiazhuang, 050011 Hebei China; 3 Department of Radiation Oncology, North China University of Science and Technology Affiliated People’s Hospital, Tangshan, 063000 Hebei China

**Keywords:** Extracellular vesicles (EVs), Ultracentrifugation (UC), Size exclusion chromatography (SEC), Filtration (ExoQuick), exoEasy, RNomics

## Abstract

**Background:**

Extracellular vesicles (EVs), known as cell-derived membranous structures harboring a variety of biomolecules, have been widely used in liquid biopsy. Due to the complex biological composition of plasma, plasma RNA omics analysis (RNomics) is easily affected, thus it is necessary to select an optimal strategy from exiting methods according to the performance for intended application.

**Methods:**

In this study, four different strategies for EVs isolation were performed and compared (i.e. ultracentrifugation (UC), size exclusion chromatography (SEC), and two most frequently-used commercially available isolation kit (ExoQuick and exoEasy). We compared the yield, purity, PCR quantification of RNAs, miRNA-seq analyses and mRNA-seq analyses of RNAs from EVs isolated using four methods.

**Results:**

The results showed that the lowest miRNA binding protein AGO2 (Argonaute-2) and the highest EVs-specific miRNA and lncRNA were observed in EVs obtained through SEC, meanwhile the content of the non-specific miRNA was the lowest. Further RNA-Seq data revealed that RNAs obtained via SEC presented more useful reads for both miRNA and mRNA. Furthermore, the mRNA delivered via SEC tended to have a concentration comparable to the ideal FPKM (Fragments Per Kilobase Million) value.

**Conclusions:**

SEC shall be used as an optimal strategy for the isolation of EVs in plasma RNomics analysis.

**Supplementary Information:**

The online version contains supplementary material available at 10.1186/s12967-021-02775-9.

## Background

Recently, increasing studies reported that circulating RNA is abundant and could be as prognosis marker. For example, serum high-temperature-required protein A2 (HtrA2) was reported to associate with the progression of breast cancer, especially could increase the diagnostic efficiency when combined with CA15-3 and CEA [1]. In addition, serum HOX transcript antisense RNA (HOTAIR) and miR-146 expression either showed the diagnostic value or could be as a sensitive maker to imatinib therapy in chronic myeloid leukemia [2, 3]. However, RNAs existing in serum are instable, thus it is limited for clinical application of RNAs. Under this condition, RNA and RNomics in extracellular vesicles (EVs) provide an potential biomarkers for diagnosis of diseases because EVs could protect RNA from degradation [4, 5]. Extracellular vesicles (EVs), with the diameter ranging from 40 to 1000 nm [6], was characteristic by protein markers (eg. TSG101, ALIX, CD63 etc.) and a variety of biomolecules, such as proteins, nucleic acids, glycans, and other signaling materials [7, 8]. EVs can be observed in almost all body fluids, and can be released by majority cells to exert various functions [9, 10]. The ability to transport biomolecules between specific cells endows EVs with application potential as targeted drug delivery carriers [11] and diagnostic markers [12–14].

Since many studies have reported that EVs could be closely related to disease progression and suggested their roles as novel biomarkers for various diseases, optimal strategy for EVs purification shall be further developed as soon as possible. Although there have been several methods available to purify the EVs, including ultracentrifugation, filtration, precipitation, chromatography, immunoaffinity capture, and commercialized kits derived from the above technologies [15–17]. However, there has been little evidence suggested that the EVs isolated by the above methods can afford downstream functional studies. At present, all techniques provide EVs recovery yield and purity of different grades. In general, impurities that cannot be separated from EVs have dimensions or densities similar to those of EVs and therefore cannot be separated in a single manner. A combination of techniques can improve the purity of the isolated EVs at the expense of a significantly reduced recovery rate. Along with the advances in medicine and biology, the diversity of biological samples and the complexity of downstream analysis also put more demands on EVs purification [18].

According to public database (http://www.exocarta.org), up to 3000 mRNAs and 2800 miRNAs have been identified in EVs. In addition, a growing number of evidence suggests that lncRNAs can be sorted by EVs [19] and affect tumorigenesis, brain disorders, and other diseases [20–22]. RNA sequencing is the most wildly used technology for EVs-based biomarker discovery in liquid biopsy, and a large number of RNA in body-fluid-derived EVs have been reported as potential diagnostic markers. In general, there could be differences in the efficiency, RNA distribution characteristics, and coverage degree of the RNA obtained by various EVs isolation methods, thus resulting in poor repeatability of the analysis results. Therefore, the performance of existing EVs extraction methods for RNA sequencing should be comprehensively compared before the research protocol is determined.

In this study, the performance of four EVs isolation methods for final usage in RNomics analysis were compared, including UC, SEC, ExoQuick and exoEasy kit. To the best of our knowledge, this study is the first research to comprehensively compared the RNomics performance of the four EVs separation methods that are the most frequently used at present. Present study might provide additional insights into the understanding of EVs isolation methods as well as basis for further investigation of EVs-based omics analysis.

## Materials and methods

### Plasma preparation

4 mL of fresh plasma (pretreatment was completed within 30 min after blood collection and the sample was subjected to EVs separation immediately without frozen) from the same donor was equally divided into 4 aliquots, each of which was subjected to one specific extraction method. Samples that come from the same donor, with the consistent pretreatment conditions, will minimize the personal equation during the experiments. The isolation of EVs was performed forth without frozen so that the impact of freezing on sample quality would be minimized. Whole blood samples were temporary stored in EDTA collection tubes at room temperature (< 30 min). Before processing, it is necessary to ensure no visible hemolysis was observed. Then, the blood sample was centrifuged at 1600 g for 15 min at room temperature to spin down cell pellet. The supernatant was transferred to a new Eppendorf tube and centrifuged at 10,000 g for 30 min to remove debris and large vesicles at room temperature. Plasma samples shall be prepared before being used.

### EVs isolation

A total of four EVs isolation methods were compared in this study: ultracentrifugation (UC), exoEasy kit (QIAGEN), exclusion chromatography (qEV [Izon], Exosupur [echobiotech]), ExoQuick kit (Thermo Fisher).

1. The EVs isolated by UC: Plasma samples were first centrifuged at 2000 g for 10 min. The supernatant was filtered using a 0.22 µm constant well filtration system (Corning, USA). The plasma were then centrifuged in a polymer centrifuge tube (Beckman Coulter, Fullerton, CA, USA) at 100,000 g and 4 °C for 2 h with a rocking rotor (Optima XPN-80, SW 55 Ti rotor, Beckman Coulter), then washed with PBS at 100,000 g and 4 °C for 2 h. The precipitate was resuspended by 1 × PBS.
2. The EVs isolated by exoEasy: The sample was centrifuged at 3000 g for 15 min at 4 °C using pre-filtered plasma. 1 portion of XBP buffer were added to the sample of 1 volume. The tube was gently inverted 5 times to mix the solution immediately and the mixture was placed at room temperature. The mixture was applied to an exoEasy spin column and centrifuged at 500 g for 1 min. The flow-through solution were discarded and the spin column was placed in the same collection tube. The residual volume in the spin column was removed by adding 3.5 mL of XWP buffer and centrifuging at 5000 g for 5 min. All flow-through solution were discarded. The spin column was moved to a new collection tube. The EVs adsorbed on column could be eluted by Buffer XE for BCA, NTA, and TEM assays, or RNA isolation by QIAzol regent.
3. The EVs isolated by SEC: SEC was performed according to the manufacturer’s instructions (QEV (Izon), Exosupur (echobiotech, China)). Briefly, plasma samples were first centrifuged at 2000 g for 10 min. The supernatant was filtered using a 0.22 µm constant well filtration system after centrifugation. Then, plasma was added on the top of the column. The EVs containing fraction were collected together by 1 × PBS. The collected fractions were concentrated by ultrafiltration with 100 K Amicon Ultra-15 centrifugal filter units (EMD Millipore).
4. The EVs isolated by ExoQuick: The plasma samples were centrifuged 3000 g for 15 min, and the supernatant was transferred to a sterile tube. The ExoQuick reagent was added, and inverted the centrifuge tube upside down several times to mix the solution well. The cells were allowed to stand at 4 °C, and then centrifuged at 1500 g for 30 min to wash the supernatant, and then centrifuged at 1500 g for 5 min and aspirated the supernatant. The precipitate was resuspended by adding 1 × PBS.

### Nano-flow cytometry (nanoFCM)

The concentration standard sample and the particle size standard sample were used for parameter calibration before the test. The data were collected for one minute by nano-flow cytometry. The particle concentration and particle diameter of the sample were determined according to the standard sample [23].

### Transmission electron microscopy (TEM)

For transmission electron microscopy, freshly isolated EVs suspensions were fixed in 4% paraformaldehyde for 1 h. The EVs suspensions from different samples (approximately 5 μL) were applied to copper mesh Formvar coated carbon stabilized grids, were allowed to adsorb to the grid for 4–5 min and then wicked off with filter paper. For negative staining of EVs, 1% aqueous uranyl acetate (5 μL) was applied to the grid for 30 s, then wicked off with Whatman filter paper. Grids were thoroughly dried before viewing [24].

### Western blot analysis

Protein samples were prepared by adding 200 μL of ice-cold NP-40 buffer with protease inhibitor to 200 μL of extracted EVs sample suspended in appropriate buffer. Mix and shake once every ten minutes then incubate on ice for 50 min. The protein concentration was measured by using BCA (Applygen, China). Then add 2 × loading buffer (200 μL), and mix well, and heat to boil. The protein sample (50 μg) was separated with an 8–10% SDS gel, and blotted on an immuno-blot PVDF membrane. Then the BSA was applied to block for 1 h and the membrane was incubated with primary antibody (APOB, AGO2, HSA, Alix, Tsg101, CD9 or CD63) overnight at 4 °C. The membrane was then washed with Tris buffered saline containing 0.1% Tween and incubated with the secondary antibody for 1 h at room temperature. Then, the film was washed again and exposed to ECL (electrochemiluminescence).

### ExoRNA isolation and RNA analyses

Total RNA was extracted and purified from plasma EVs using miRNeasy® Mini kit (Qiagen, cat. No. 217004) according to the kit instruction. RNA degradation and contamination, especially DNA contamination, was monitored on 1.5% agarose gels. RNA concentration and purity were evaluated using the NanoDrop 2000 Spectrophotometer (Thermo Fisher Scientific, Wilmington, DE) and the RNA Nano 6000 Assay Kit of the Agilent Bioanalyzer 2100 System (Agilent Technologies, CA, USA). All the RNA sequencing was performed using the single sample.

### Library preparation and sequencing

A total of 5 ng RNA per sample was used as input material for sequencing libraries using the Ovation® SoLo RNA-Seq Library Preparation Kit (NuGEN, CA, USA) following manufacturer’s recommendations and index codes were added to attribute sequences to each sample. For small RNA libraries, a total of 2.5 ng RNA per sample was used as input material for the RNA sample preparations. Sequencing libraries were generated using NEB Next Multiplex Small RNA Library Prep Set for Illumina (NEB, USA) following manufacturer’s recommendations and index codes were added to attribute sequences to each sample. At last, PCR products were purified (AMPure XP system) and library quality was assessed on the Agilent Bioanalyzer 2100 and qPCR. The clustering of the index-coded samples was performed on acBot Cluster Generation System using TruSeq PE Cluster Kitv3-cBot-HS (Illumia) according to the manufacturer’s instructions. After cluster generation, the library preparations were sequenced using an Illumina Hiseq platform and paired-end reads were generated [25, 26].

### Statistical analysis

Comparison of the variable average was performed using SPSS 19.0 statistical software. Data were plotted as mean ± SEM. GraphPad Prism was used for statistical analysis and graph generation (GraphPad Software, San Diego, USA). After testing the normality and the same variance using the Shapiro–Wilk and Levene tests, the difference between the abnormal averages was analyzed by using the Kruskal–Wallis test. A P value less than 0.05 was considered to be statistically significant.

## Results

### Comparison of the yield and purity of EVs isolated by four methods

**Fig. 1:**
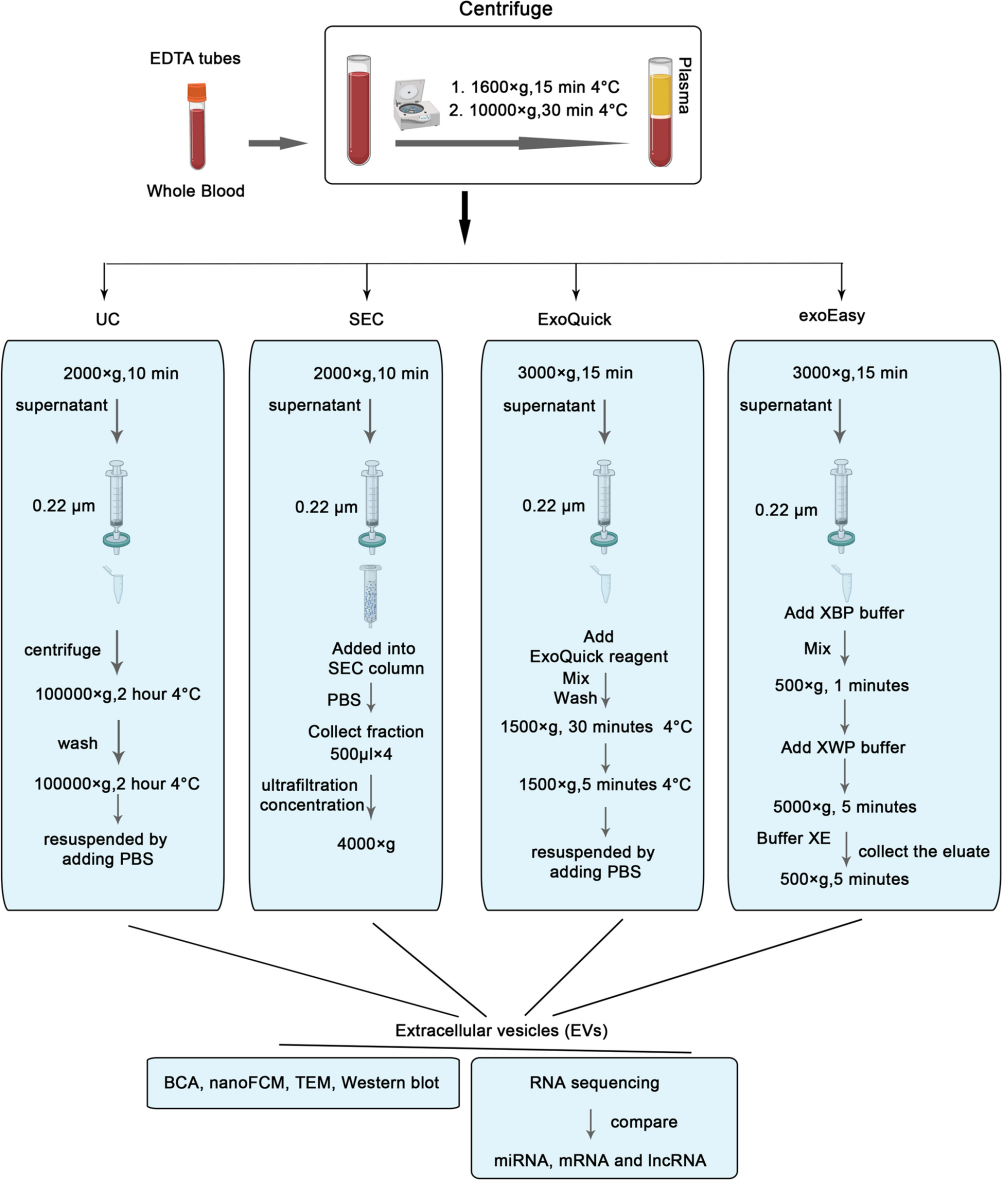
Experimental design model diagram: Whole blood were collected from healthy people in EDTA tubes, and the plasma were separated from the whole blood through centrifugation. EVs were separated from plasma by four methods SEC, exoEasy, UC or ExoQuick respectively. Then the isolated EVs were used for RNA sequencing and Western blotting experiments, etc.

In order to find out the optimal isolation method for RNomics from EVs, we compared four existing methods, including UC, SEC, exoEasy, and ExoQuick. The whole experimental design and the operation procedures was shown in Fig. 1. A total of three SEC-based EVs isolation kits were tested for EVs purification (qEV (Izon), Exosupur (echobiotech) and 4ff). An artificial EVs mimic (fluorescently-labeled liposome with an average diameter of about 100 nm) was added into the plasma and the EVs containing fractions were collected for further detection of fluorescence intensity and protein concentration. According to our results, the fractions collected from Exosupur showed the highest fluorescence intensity (Additional file 1: Fig. S1A) and lowest protein concentration (Additional file 1: Figure S1B and C), indicating the most effective EVs recovery rate and lowest contamination of free protein. Further TEM and immunoblotting results confirmed the enrichment of EVs in the collected fractions (Additional file 1: Figure S1D and E). Therefore, Exosupur was used for further horizontal comparison experiments.

**Fig. 2:**
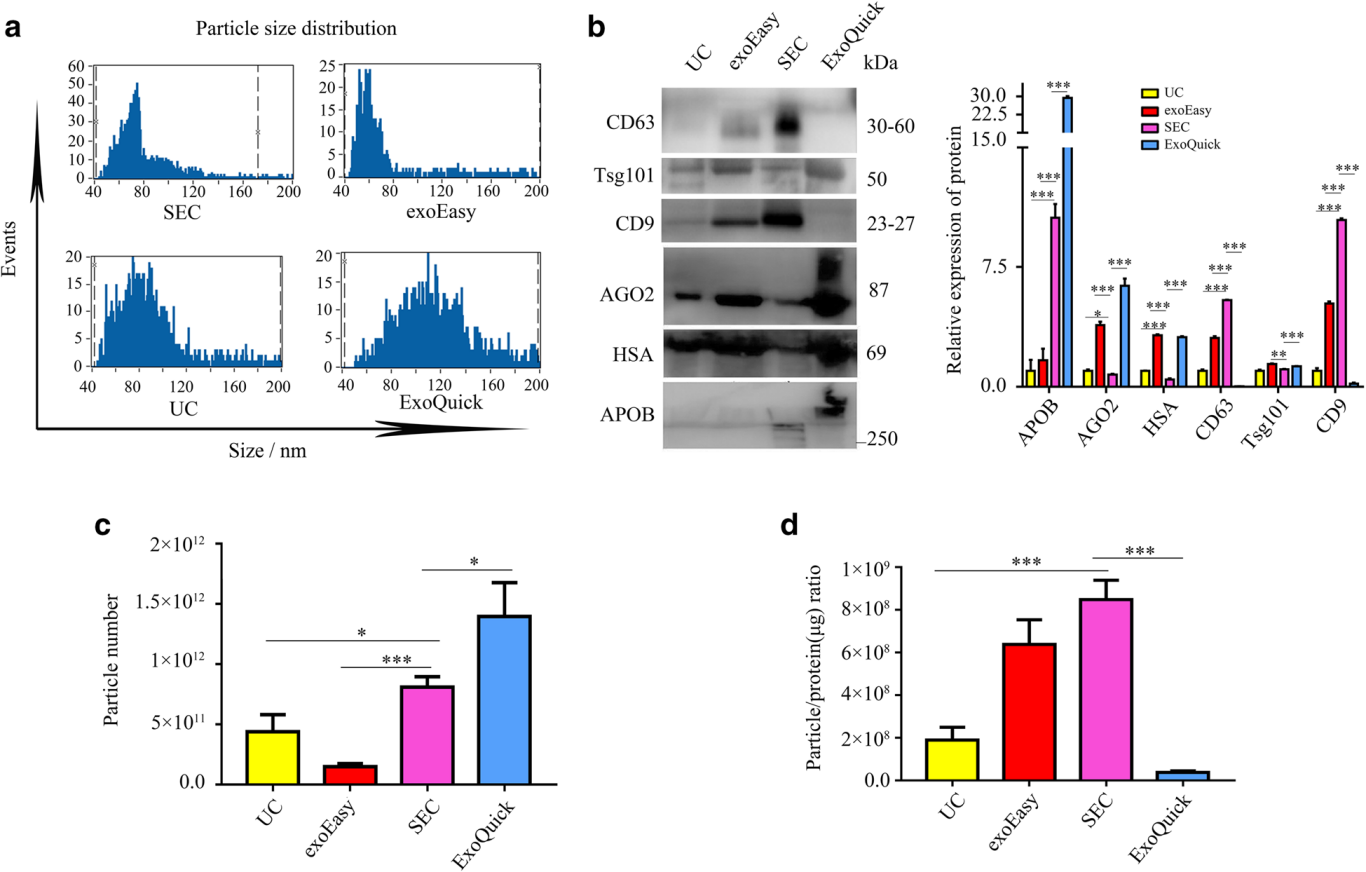
EVs yield and purity isolated by SEC, exoEasy, UC and ExoQuick methods. **a** Particle size distribution of EVs isolated by Exosupur, exoEasy, UC and SBI analyzed by nano-flow cytometry (nanoFCM). **b** The EVs protein expressions of APOB, AGO2, CD63, Tsg101, CD9 and HAS isolated by SEC, exoEasy, UC and ExoQuick(left). Quantified band intensity data of the EVs protein(right). **c** Particle number in SEC, exoEasy, UC and ExoQuick samples measured by nano-flow cytometry. **d** Particle/protein ratio for SEC, exoEasy, UC and ExoQuick samples. *P < 0.05, **P < 0.01, ***P < 0.001

Bradford protein assays (BCA) and nano-flow cytometry (nanoFCM) were applied to analyze the total amount of protein, particle concentration, and size distribution of the EVs products obtained via UC, SEC, exoEasy and ExoQuick. As showed in Fig. 2a, nanoFCM analyses presented that the EVs obtained by ExoQuick were larger than 100 nm, while the EVs achieved by the other three methods (UC, SEC, exoEasy) were smaller than 100 nm. Moreover, the EVs extracted by the four methods all expressed EVs-specific protein markers including CD63, Tsg101 and CD9 (Fig. 2b), in spite of the slight differences in particle size distribution of EVs. And the protein expression of AGO2, HSA, and APOB were highest in the EVs isolated by ExoQuick, while the protein expression of HSA and AGO2 were lowest in the EVs isolated by SEC (Fig. 2b). In addition, nanoFCM analyses also suggested that the numbers of the particles isolated by ExoQuick, SEC, UC, and exoEasy were in a descending order (Fig. 2c). Since co-isolated impurities may contain protein components, the particle/protein ratio of each individual method was examined. According to the result, SEC had the highest particle/protein ratio, while the order of values for the rest three methods was exoEasy > UC > ExoQuick (Fig. 2d). Taken together, it was suggested that ExoQuick might have the highest particle recovery rate, while SEC can achieve the most satisfactory purity, with the lowest content of EVs-free RNA binding protein.

### PCR quantification of RNAs extracted from EVs isolated by different methods

**Fig. 3:**
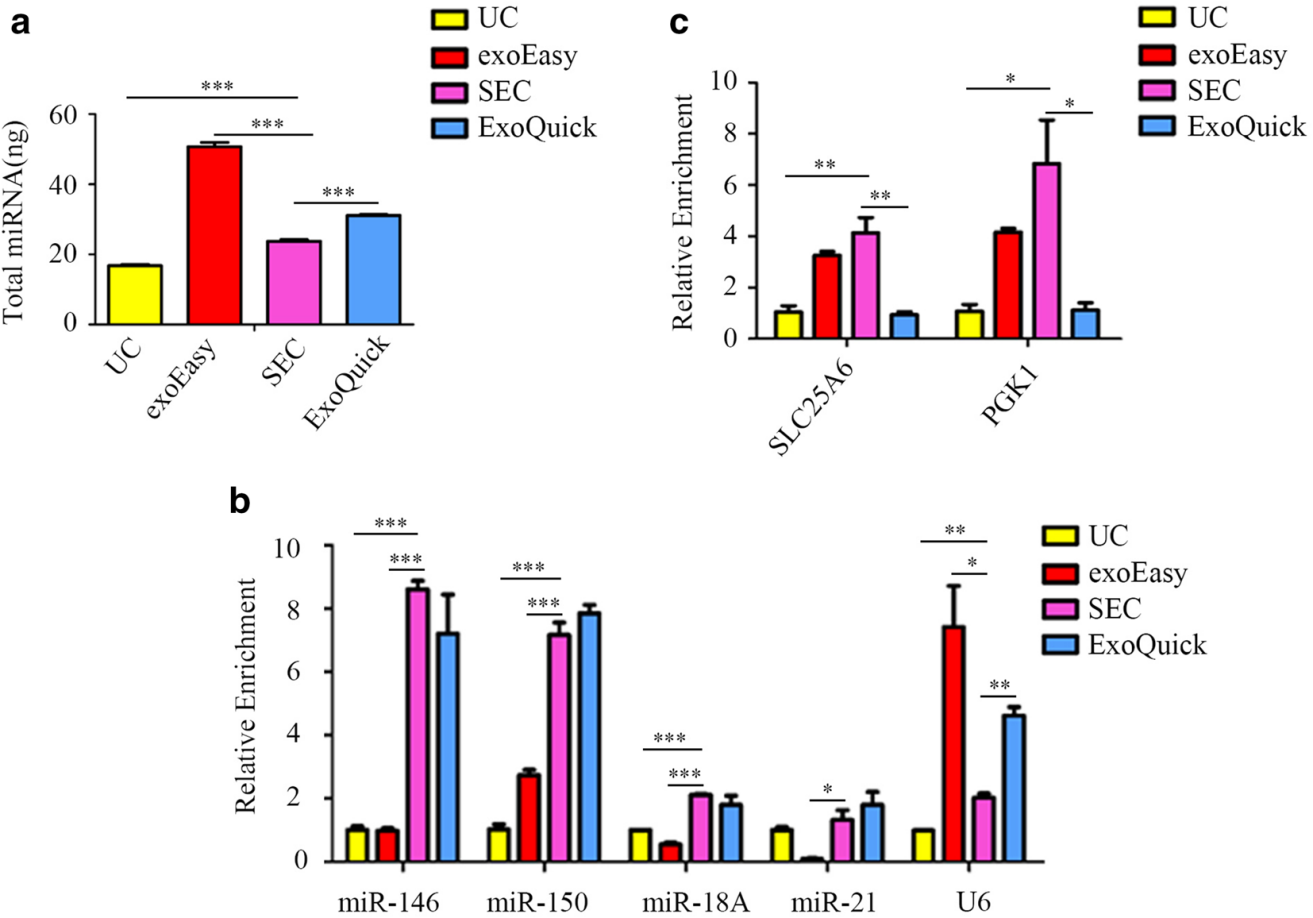
Characterization of RNAs extracted from EVs isolated by SEC, exoEasy, UC and ExoQuick respectively. **a** Total miRNAs in RNAs obtained from EVs. **b** qRT-PCR analyses of EVs-specific miRNAs (miR-146, miR-150, miR-18A and miR-21) and free miRNAs (like U6). **c** qRT-PCR analyses of EVs-specific lncRNAs (SLC25A and PGK1). *P < 0.05, **P < 0.01, ***P < 0.001

To serve the purpose of RNomics, several characteristics of RNA products need to be evaluated, such as quantity, purity, types of RNA and so forth. As showed in the Fig. 3a, total miRNA quantities, which represented the total RNA quantities here, were measured. The results showed that the amounts of the total RNA in the collected fractions isolated by exoEasy, ExoQuick, SEC, and UC were in descending order. However, the total amount of enriched miRNAs was insufficient to reflect the superiority for RNomics. More than 90% of the miRNAs in plasma were EVs-free and may bind to AGO2 protein. Therefore, the collected miRNA may also contain mass EVs-free miRNAs. It has been reported by previous studies that microRNAs can exhibit differentiated distribution schema in plasma. For example, miR-146 [27], miR-150 [28] and miR-18A [29] tends to be associated with vesicles in plasma, while miR-21 [30] and U6 [31] are more likely to present in non-vesicle form (e.g., RNA binding protein or complex). Thus, the relative abundance of these vesicle-enriched and non-enriched microRNAs can be used to reflect the relative quantity and purity of EVs-specific RNAs, as well as the purity of EVs products. As shown in Fig. 3b, qRT-PCR results suggested that the EVs isolated by SEC had relatively higher amount of miR-146, miR-150, miR-18A and less amount of miR-21 and U6, indicating the method may achieve the highest content of miRNAs derived from vesicles and lowest amount of non-vesicle miRNAs. Apart from microRNAs, the abundance of long-chain RNA molecules extracted from EVs products was also investigated. Unlike microRNA, extracellular long-chain RNA molecules can hardly survive without the protection from vesicle membrane [32]. As such, the abundance of two mRNA molecules (SLC25A and PGK1) in the EVs isolated by different approaches were measured. As showed in Fig. 3c, the relative enrichment of both SLC25A and PGK1 in the fractions isolated by SEC was remarkably higher than that of the products purified by other methods. All these results demonstrated that the EVs obtained via SEC may provide a higher yield and more promising purity regarding EVs-specific RNAs.

### miRNA-seq analyses of RNAs extracted from EVs isolated by different methods

**Fig. 4:**
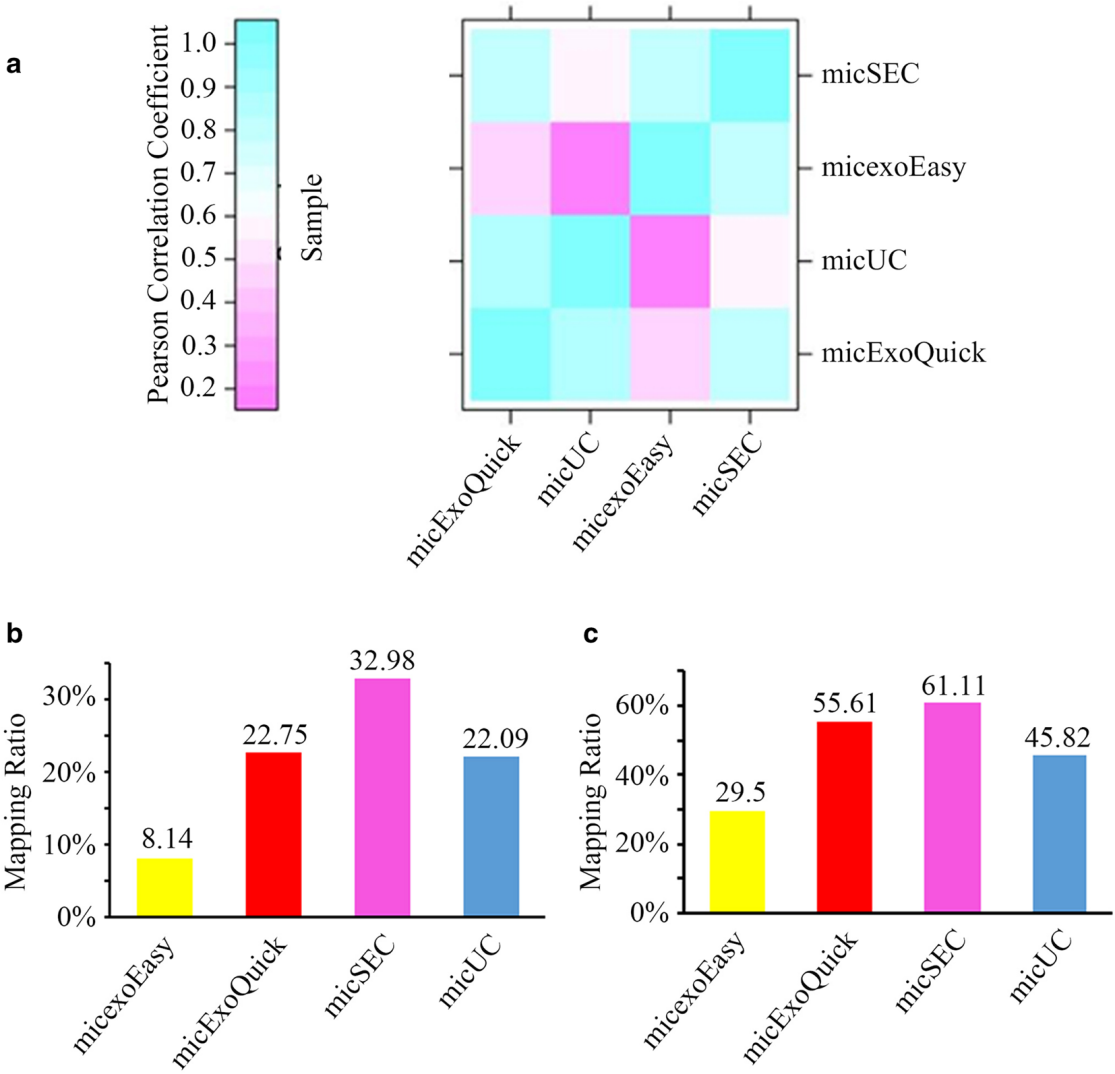
Characterization of small RNAs (18–32 bp) identified by RNA-seq. **a** Heatmap representing the consistency of the profiles of small RNAs obtained via the four EVs isolation methods. **b** Percentage of miRNA reads identified by database miRbase (V22) and software miRDeep2 (exoEasy: 8.14%, ExoQuick: 22.75%, SEC: 32.98%, UC: 22.09%). **c** Percentage of mapped reads based on the reference genome (exoEasy: 29.5%, ExoQuick: 55.61%, SEC: 61.11%, UC: 45.82%)

In order to investigate the expression profile of different types of RNAs, the properties of RNA sequencing in the EVs isolated by the four approaches were examined. The miRNA sequencing was performed to comprehensively evaluate the characteristics of RNA products. According to the consistency results in Fig. 4a, there were obvious differences in the RNA profiles generated by different isolation methods, and the miRNA profile of the exoEasy showed the largest deviation compared with the other three methods. This difference might be aroused by the innate bias as each individual method targets at distinct biochemical properties of EVs. Of the total sequencing reads, SEC-derived RNA showed the highest percentage of miRNA-specific reads (18–32 nt), followed by UC and exoEasy (Fig. 4b). Although ExoQuick enriched large amount of RNAs and proteins, the lowest percentage of miRNA reads was recorded, which was less than 10%. Besides, the percentages of the miRNA reads were compared with human miRNA database. The results suggested that more than 50% of the miRNA reads in SEC could be mapped to human miRNA database (Fig. 4c). All these data indicated SEC-derived RNA showed the highest percentage of useful miRNA sequencing data for further biomarker identification.

### mRNA-seq analyses of RNAs extracted from EVs isolated by different methods

**Fig. 5:**
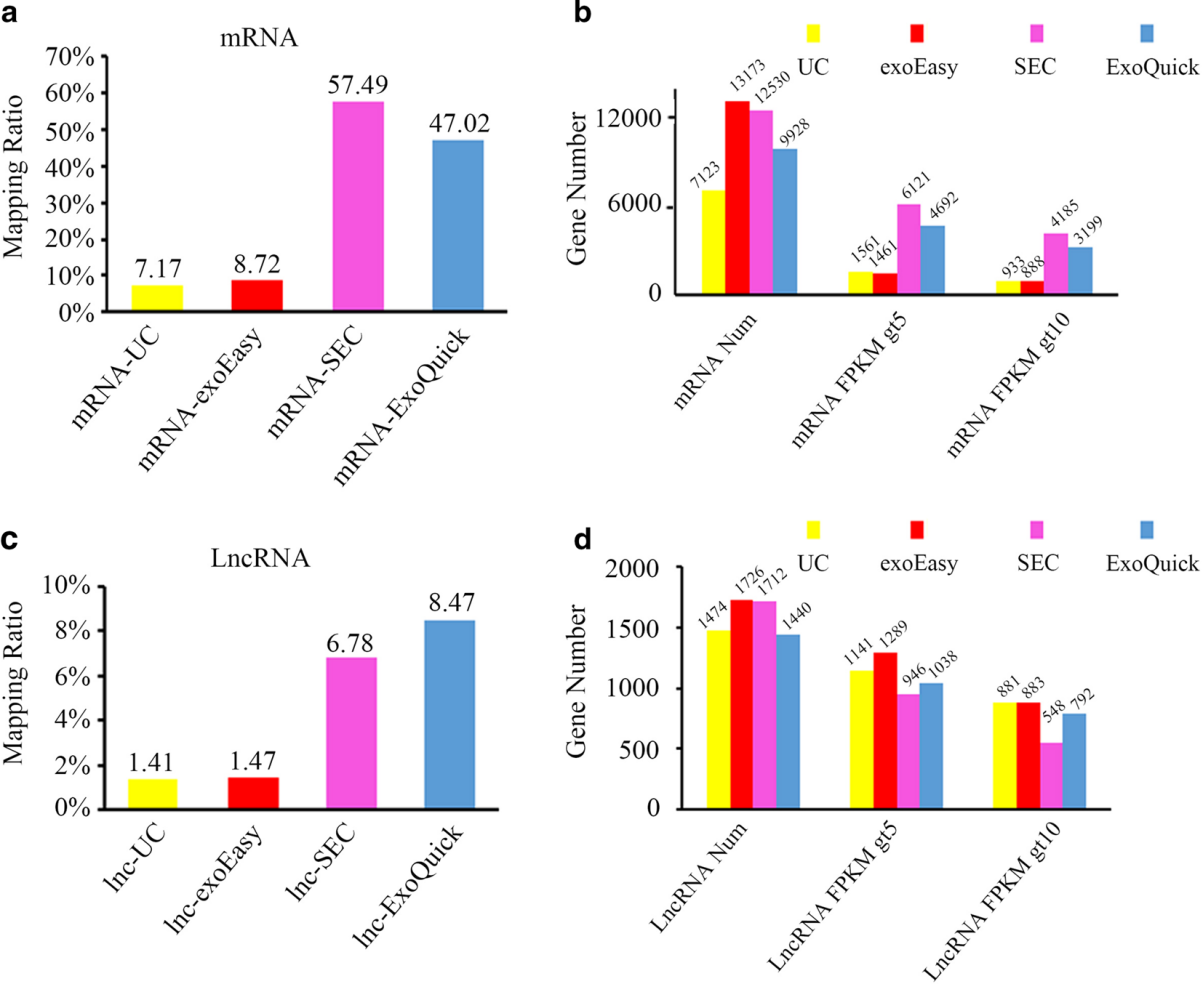
Characterization of lncRNAs and mRNAs in long RNAs. **a** and **c** Percentage of mRNA reads and expression analysis using String Tile. **b** and **d** Percentage of lncRNA reads and expression analysis using String Tile. FPKM: Fragments per kilobase of transcript per million fragments mapped

RNA sequencing analysis was also performed with the long chain RNAs (mRNA and lncRNA). As shown in Fig. 5a, SEC and ExoQuick achieved the highest mRNA reads count (UC: 7.17%, exoEasy: 8.72%, ExoQuick: 47.02%, SEC: 57.49%). Similar tendency was also observed in the results of lncRNA (UC: 1.41%, exoEasy: 1.47%, ExoQuick: 8.47%, SEC: 6.78%) (Fig. 5b). The numbers of genes in mRNA sequencing data were compared as well. The largest amount of mapped mRNA transcripts was achieved via SEC and exoEasy. Further analysis showed that although abundant mRNA transcripts were detected in exoEasy, majority of the mRNA transcripts showed a FPKM of less than 5. This tendency suggested the low abundance of most of the mRNA detected by exoEasy in mRNA sequencing, which may increase the difficulty in further PCR verification. In contrast, mRNA sequencing in SEC-purified mRNA not only provided the highest abundance of useful mRNA reads, but also detected maximum kinds of mRNA with considerable expressive abundance (mRNA with FPKM > 5 or FPKM > 10) (Fig. 5c).

Regarding lncRNA, there was little difference in the types of the total mapped lnRNA and lncRNAs with a FPKM > 5 among the four methods since most of the reads in long chain RNA sequencing belonged to mRNA and the total reads mapped to lncRNA were not enough for effective comparison (1.41–8.47%) (Fig. 5d).

### Characterization of long chain RNAs identified by RNA-seq

**Fig. 6:**
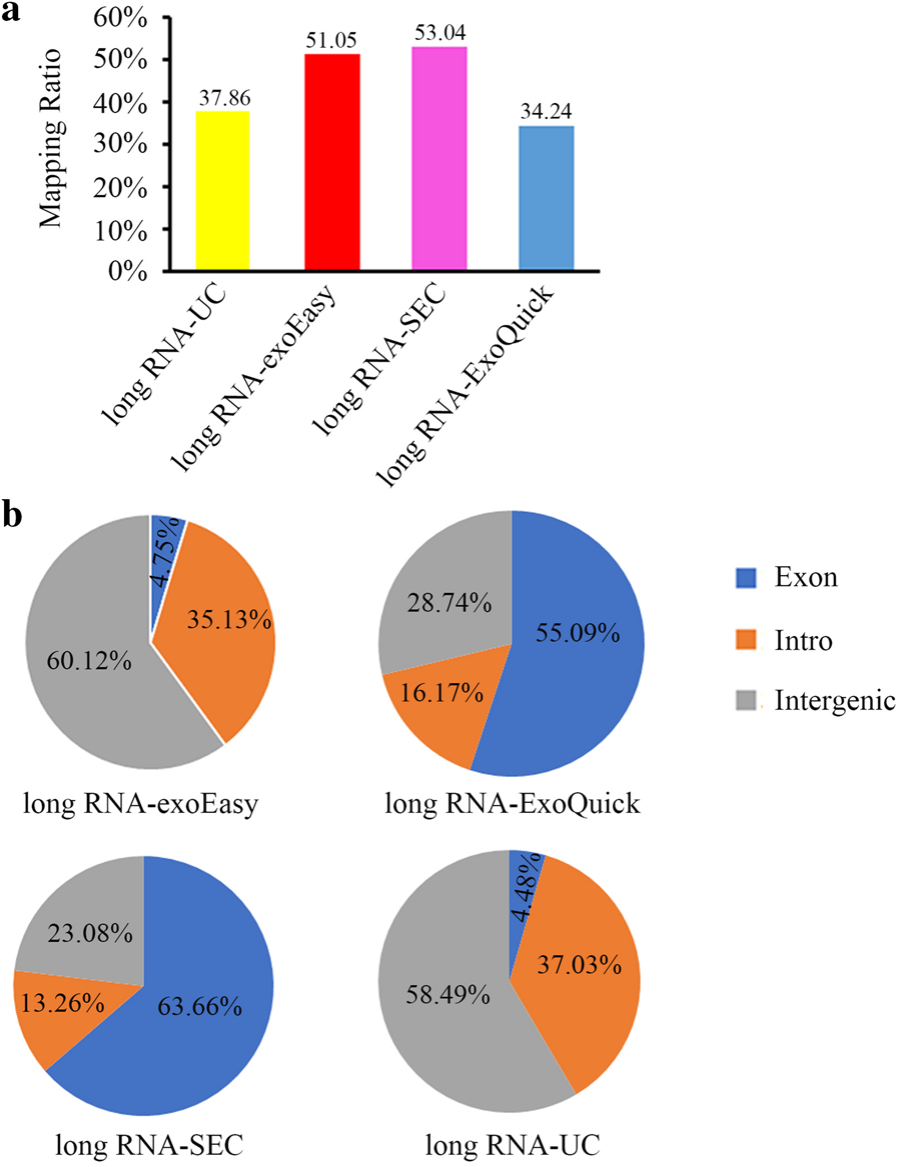
Characterization of long chain RNAs identified by RNA-seq. **a** Percentage of mapped long chain RNA reads based on the reference genome GRCh38. **b** Percentage of exon, intron and intergenic region in mapped long chain RNA sequencing

When assessing the sequencing results of long chain RNAs, SEC and exoEasy displayed similar mapping rates (53.04% vs. 51.05%), while the values of UC and ExoQuick were 37.86% and 34.24%, respectively (Fig. 6a). However, the pie chart in Fig. 6b suggested that, of all these mapped reads, the exon rate of SEC was much higher than that of exoEasy (UC: 4.48%, exoEasy: 4.75%, ExoQuick: 55.09%, SEC: 63.66%). Nevertheless, most of the mapped reads in exoEasy and UC were enriched in intron and intergenic region. As the intron and intergenic should not be present in mature mRNA/lncRNA, the percentage of reads mapped to exon could reflect the percentage of mature mRNA. This probably can be attributed to the fact that the electric-charge-based exoEasy will co-isolate the circulating cell free DNA (cfDNA) in plasma. It is worthy to mention that, for the purpose of mutation analysis, co-isolation of both cfDNA and vesicle enclosed RNA gives additional copies of target gene associated nucleic acid, which would increase the detection sensitivity for mutations with low frequency comparing to simply analyzing cfDNA.

## Discussion

In recent years, accumulating evidences had noted the importance of RNA or proteins obtained from EVs as potential clinical diagnostic markers and therapeutic strategy [33–35]. Along with the advanced understanding of EVs, there have been more and more requirements regarding the purification of EVs. Nowadays, UC, SEC, exoEasy, and ExoQuick are the most frequently-used methods to extract the EVs, while different method presented varied advantages regarding purity, recovery rate, and selectivity of proteins and RNAs [36]. The EVs isolation method with stable, reliable property for RNA sequencing is indispensable for the clinical translational study of EVs. However, there has been no literature reporting the comprehensive comparison among the four wildly-used EVs purification methods in RNA sequencing untill now.

In terms of the purity and recovery rate, the results suggested that the property of SEC-derived EVs was variable, which was related to the kit selection and the fractions collected in the experiment. When using SEC for further EVs-related analysis, it is of vital importance to choose a reliable SEC column. It was reported that majority of the RNA in plasma were not packaged by EVs, but presented as a complex with RNA binding proteins (e.g., HAS and AGO2). The abundance of such proteins could reflect the influence of EVs free RNAs in further RNA sequencing work. Therefore, in present study, the contents of potential protein contamination markers were evaluated. As showed in Fig. 2b, the isolated product of ExoQuick contained a large amount of soluble plasma protein, such as AGO2, HSA, and APOB, while SEC was confirmed to have the lowest contamination of HSA and AGO2. It’s worth noting that SEC did show a higher amount of APOB protein comparing to the UC and exoEasy, which was consistent with other studies. The reason for this phenomenon could be that the size of the low-density protein (LDL) is similar to that of EVs. Therefore, ultracentrifugation could be applied to separate the EVs and LDL in the collected fractions by means of the difference in density (LDL has a lower density of < 1 gmL^−1^).

According to the protein concentration and PCR quantification assay, SEC-purified RNA showed a better vesicle enrichment characteristic. As the majority of miRNAs in plasma are AGO2 associated rather than EVs’ cargoes, SEC could wipe off free proteins efficiently and ensure that most of the miRNAs in further sequencing are EVs-specific. However, both SEC and exoEasy have the ability to exclude AGO2 protein, which might cause the miRNA detected in sequencing are not EVs-specific. Furthermore, in long chain RNA sequencing, most of the mapped reads in SEC enriched RNA were located in exon, indicating less influence from cfDNA or other non-classical long chain mRNA in plasma. Although SEC did not show outstanding advantage with respect to the total number of mRNA kinds in RNA sequencing, the detected mRNAs were preferring to be with considerable concentration with ideal FPKM values. This suggested differential expressed mRNAs in SEC based RNA sequencing will showed better reproducibility in further PCR validation work.

Though RNA sequencing in plasma sample was the most widely concerned research area in liquid biopsy, differential EVs extraction method has different efficiency. RNA distribution characteristics and coverage in RNA sequencing make the analysis results showing inadequate repeatability in different groups. For the first time we provided the RNomics panorama including transcriptome sequencing of mRNA, miRNA and lncRNA of all the used EVs extraction methods, which will provide important reference value for plasma EVs based on RNA sequencing work. Moreover, we not only proved SEC based EVs isolation was most superior for RNA sequencing, but also supplied an artificial fluorescently-labeled liposome to evaluate the merit advantage of SEC column in EVs isolation. This system will supply important reference for the SEC based EVs research.

For the first time we evaluated four EVs isolation methods comprehensively in RNomics including miRNA, mRNA and lncRNA. This is the most systematic work that comparing EVs isolation methods in the respect of RNomics. Other researches on the comparison of RNA extracted from EVs, were that comparison of miRNA and mRNA from urinary extracellular vesicle extracted by Hydrostatic Filtration Dialysis (HFD), ultracentrifugation (UC), and a commercial kit‐based isolation method (NG) [37], or comparison of miRNA from plasma samples EVs of healthy dogs by ultracentrifugation, precipitation, and membrane affinity chromatography methods [38], or comparison of miRNA from plasma extracellular vesicles of HIV/HCV coinfected patients by ultracentrifugation and precipitation methods [39], or comparison of total RNA and mRNA from Whole Blood EVs extracted by only two methods including ultracentrifugation (UC) and Exodisc, a fully integrated centrifugal microfluidic device, which has previously been demonstrated to enrich EVs from urine and cell-culture supernatant [40]. In addition, few studies report the comparison of extraction exosomal lncRNA by these four different methods SEC, exoEasy, UC and ExoQuick.

In summary, it is recommended that SEC shall be used for the RNA sequencing works in EVs. However, it is worth noting that the conclusion shall not be extended to the EVs-related proteomics work in plasma, as RNA and protein have variant distribution pattern. Furthermore, due to the similar particle sizes of LDL, HDL, and EVs, it is inevitable to introduce LDL/HDL to the EVs fractions during SEC process.

## Conclusions

In this study, we compared four different methods (i.e. ultracentrifugation (UC), size exclusion chromatography (SEC), and two most frequently-used commercially available isolation kit (ExoQuick and exoEasy)) in the isolation of EVs for RNomics analysis in plasma comprehensively. The results showed that EVs obtained by SEC had the minimum miRNA binding protein AGO2 and the highest amounts of EVs specific miRNA and lncRNA, but the lowest nonspecific miRNA. Further RNA-Seq data revealed that RNAs from SEC presented more useful reads for both miRNA and mRNA. Furthermore, the detected mRNA in SEC delivered RNA were preferring to be with considerable concentration with ideal FPKM values. In conclusion, we recommend SEC for EVs RNomics in plasma.

## Supplementary Information

**Additional file 1: Fig. S1.** EVs isolated by SEC. A. Particle fluorescence value in SEC fractions 0.5–11.5 ml, which can represent particle number. B. Protein amount and particle number of EVs isolated by kits Exosupur, QEV and 4ff. Protein was measured by BCA assays and particle number by detection of A480. C. Particle/protein ratio for EVs isolated by kits Exosupur, QEV and 4ff. Exosupur Kit was chosen because of its high particle/protein ratio. D. TEM images of Exosupur fractions 1.5 and 2.0 ml from plasma, urine and supernatant of cell culture. E. Western blotting analysis of specific markers Alix, Tsg101, CD63 and inspecific marker Calnexin on cell lysates and EVs lysates from plasma, urine and supernatant of cell culture. CL: Cell lysates as control.

## Data Availability

The datasets used or analyzed during the current study are available from the corresponding author on reasonable request.

## Abbreviations

EVs: Extracellular vesicles
UC: Ultracentrifugation
SEC: Size exclusion chromatography
ECL: Electrochemiluminescence
AGO2: Argonaute-2
FPKM: Fragments Per Kilobase Million
BCA: Bradford protein assays
nanoFCM: Nano-flow cytometry
