# Supplementary figures and images for: Extracellular vesicles isolated by size-exclusion chromatography present suitability for RNomics analysis in plasma
Source: J Transl Med. 2021 Mar 12;19:104. doi: 10.1186/s12967-021-02775-9 (PMC7953782; doi:10.1186/s12967-021-02775-9)

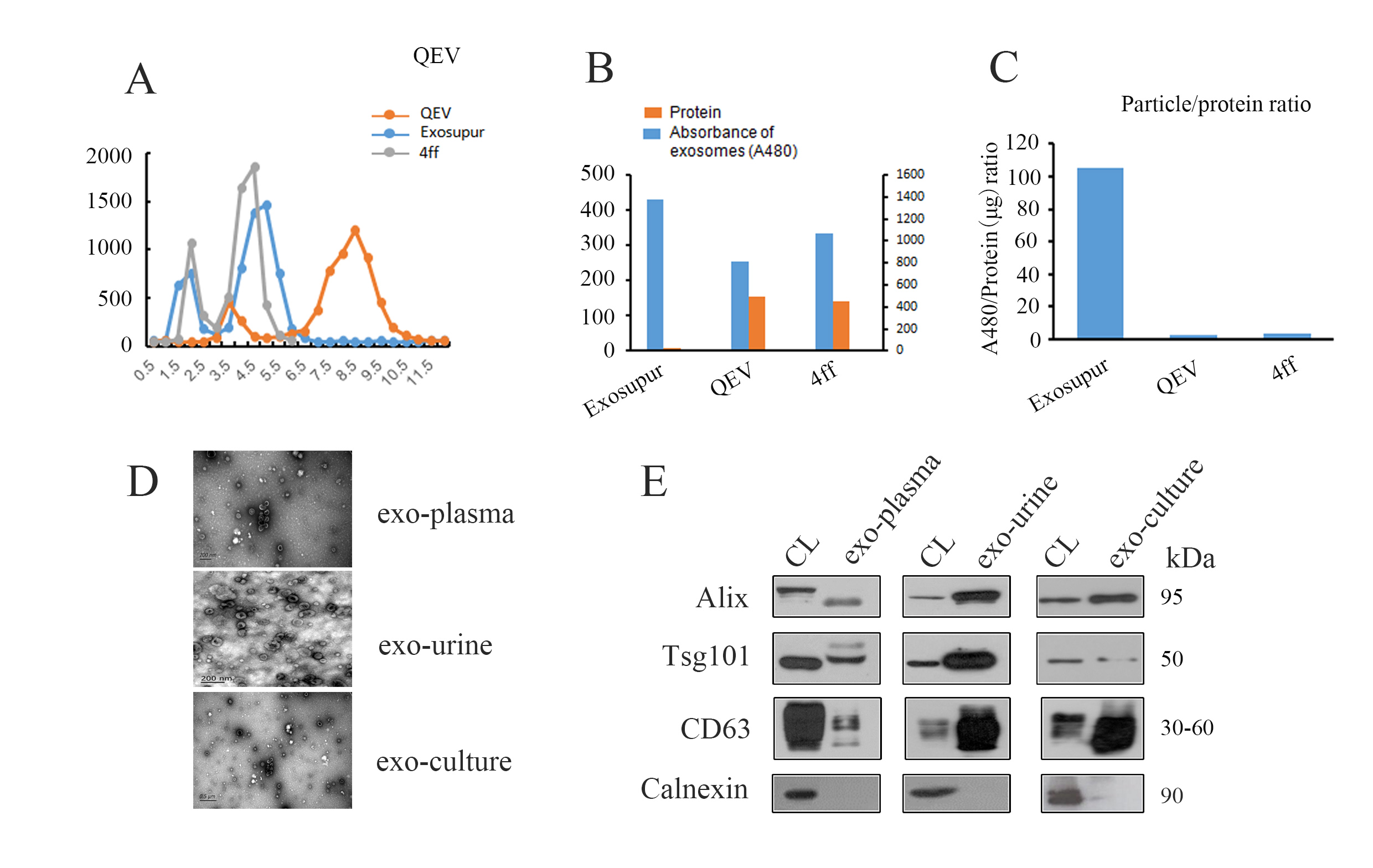

Supplement: Supplementary file 1 — Additional file 1: Fig. S1. EVs isolated by SEC. A. Particle fluorescence value in SEC fractions 0.5–11.5 ml, which can represent particle number. B. Protein amount and particle number of EVs isolated by kits Exosupur, QEV and 4ff. Protein was measured by BCA assays and particle number by detection of A480. C. Particle/protein ratio for EVs isolated by kits Exosupur, QEV and 4ff. Exosupur Kit was chosen because of its high particle/protein ratio. D. TEM images of Exosupur fractions 1.5 and 2.0 ml from plasma, urine and supernatant of cell culture. E. Western blotting analysis of specific markers Alix, Tsg101, CD63 and inspecific marker Calnexin on cell lysates and EVs lysates from plasma, urine and supernatant of cell culture. CL: Cell lysates as control. [file 12967_2021_2775_MOESM1_ESM.jpg]
